# Pathway Tools Management of Pathway/Genome Data for Microbial Communities

**DOI:** 10.3389/fbinf.2022.869150

**Published:** 2022-04-26

**Authors:** Peter D. Karp, Suzanne Paley, Markus Krummenacker, Anamika Kothari, Michael J. Wannemuehler, Gregory J. Phillips

**Affiliations:** ^1^ Bioinformatics Research Group, Artificial Intelligence Center, SRI International, Menlo Park, CA, United States; ^2^ Department of Veterinary Microbiology, Iowa State University, Ames, IA, United States

**Keywords:** microbiome, data management, genome database, metabolic pathways, metabolic routes

## Abstract

The Pathway Tools (PTools) software provides a suite of capabilities for storing and analyzing integrated collections of genomic and metabolic information in the form of organism-specific Pathway/Genome Databases (PGDBs). A microbial community is represented in PTools by generating a PGDB from each metagenome-assembled genome (MAG). PTools computes a metabolic reconstruction for each organism, and predicts its operons. The properties of individual MAGs can be investigated using the many search and visualization operations within PTools. PTools also enables the user to investigate the properties of the microbial community by issuing searches across the full community, and by performing comparative operations across genome and pathway information. The software can generate a metabolic network diagram for the community, and it can overlay community omics datasets on that network diagram. PTools also provides a tool for searching for metabolic transformation routes across an organism community.

## 1 Introduction

The Pathway Tools (PTools) software Karp et al. (2019), Karp et al. (2020) was originally developed to facilitate functional analysis of individual genomes. The software has a range of capabilities including genome informatics, metabolic pathway informatics, regulatory informatics, omics data analysis, and comparative analysis. A typical workflow is to import a genome into PTools, compute a metabolic reconstruction, infer operons of the organism, and then apply the search, visualization, and comparative analysis tools to investigate the functional properties of the organism.

The software has been extended in recent years to support functional analysis of microbiomes (Prakash and Taylor, 2012; Krishnan et al., 2015; Sung et al., 2017; Eng and Borenstein, 2019; Visconti et al., 2019) that provides causal insights regarding the interactions of organisms within a microbiome. Whereas many microbiome-related informatics tools aim to quantify and compare properties of an overall community, PTools is more focused on enabling detailed reconstructions of community members and their interactions. For example, PTools does not perform taxonomic analysis of metagenome samples, nor does it compute case-control studies such as statistical comparison of healthy and diseased individuals.

Questions that can be addressed using the software include the following: What metabolic reactions and pathways are present in a metagenome, or in each organism in a community? How do their metabolic capabilities complement one another? What pathways are unique to a given community member? What metabolic transformations can be accomplished by the community, for example, via what metabolic route might the community convert a starting metabolite into an ending metabolite? The PTools software does perform metabolic modeling of individual microbes and of microbial communities via flux-balance analysis Latendresse et al. (2022) (see also Greenblum et al., 2013; Levy and Borenstein, 2014; Krishnan et al., 2015; Esvap and Ulgen, 2021; Heinken et al., 2021), although that topic is beyond the scope of this article.

The first step in a typical workflow is to import a set of metagenome-assembled genomes (MAGs) into PTools. Each MAG is converted to a PTools Pathway/Genome Database. A number of PTools computational inference tools are next applied to each MAG to infer its metabolic reactions and pathways, its transport reactions, and its operons. The community members are now captured within a set of PGDBs that comprehensively encode their genomes and metabolic networks.

Next the user can apply a set of search and comparative analysis tools to assess and compare the functional capabilities of community members. For example, the user can search across all community PGDBs for the presence of a gene, a metabolite, or a pathway; the software can produce comparisons of the entire metabolic networks of the community.

If meta-transcriptomics and/or meta-metabolomics data are available for the community, then PTools provides an analysis tool for visualizing such data on a multi-organism metabolic map diagram.

PTools provides a community route-search tool that requires as user inputs a set of PGDBs as well as a starting metabolite and ending metabolite. The tool generates minimal-cost metabolic routes (linear reaction paths) from the starting to the ending metabolite that show how the community might accomplish that transformation.

The remainder of the article describes these tools in more detail and illustrates their use on the Altered Schaedler Flora (ASF), a community of eight microorganisms from the mouse gut microbiome Wannemuehler et al. (2014). The ASF were selected by experimentalists as a model microbiome for their dominance and persistence in the mouse gut, and for their ability to be grown in the laboratory.

## 2 Methods

### 2.1 Importing a Microbial Community Into Pathway Tools

To import a microbial community into PTools, the metagenomic sequencing data must have been binned by a separate program into separate groups, one for each detected member of the community. Each such MAG consists of a collection of sequenced contigs covering a subset of the genome of each organism. The contigs must be annotated by a tool such as MetaPathways Konwar et al. (2013), MetaErg Dong and Strous (2019), MG-RAST Keegan et al. (2016), MEGAN Huson et al. (2016), Prokka Seemann (2014), or the National Center for Biotechnology Information (NCBI) Prokaryotic Genome Annotation Pipeline Tatusova et al. (2016), meaning that an ORF-finding program has been run on each organism, and protein function-prediction tools have been run on each identified gene to assign protein names such as “pyruvate kinase,” as well as to assign Enzyme Commission (EC) numbers (optional).

The resulting sequence data, gene locations, and protein functions can be provided as inputs to PTools in either GFF3 format or GenBank format, preferably as one file per MAG. The files can be provided within a directory structure containing one directory per genome that is processed by invoking the PathoLogic component of PTools from the command line, as described in the Pathway Tools User’s Guide SRI International (2021).

PathoLogic applies a series of processing steps to each input MAG to obtain a comprehensive PGDB for each organism. Those steps are as follows.

1. The input files are parsed.
2. The input sequence, gene locations, and annotations are converted to PGDB format. PGDBs are encoded using the Ocelot object-oriented database system. A database object is created for each replicon, each gene, and each gene product described by the input files.
3. The reactome of the organism is predicted from the annotated gene functions using a previously published algorithm Karp et al. (2011). Enzyme names and EC numbers are associated with biochemical reactions via queries to the MetaCyc metabolic database (DB) Caspi et al. (2020). Those reactions are imported into the new PGDB from MetaCyc.
4. The metabolic pathways of the organism are predicted from the predicted reactome Karp et al. (2011). For each pathway in MetaCyc the prediction algorithm considers which of its component reactions are catalyzed by an enzyme in the PGDB, and computes a score expressing the likelihood that the pathway is present. Pathways that exceed a threshold are imported into the PGDB.
5. The Transport Inference Parser Lee et al. (2008) is executed to predict the transport reactions of the organism from annotated transporter names.
6. The PTools operon predictor Romero and Karp (2004) is executed to predict the operons of the organism.
7. PathoLogic executes an automatic layout algorithm that creates an organism-specific metabolic network diagram for the organism based on its complement of pathways, metabolic reactions, and transport reactions Paley et al. (2021).

The result of this process is a community of PGDBs—one for each binned organism—describing its genome, proteome, reactome, metabolic pathways, and operons. For example, we have created PGDBs for each of the eight members of the ASF, all of which are available through the BioCyc.org website (which is powered by PTools). Enter “ASF” into the BioCyc organism selection tool to search for these databases.

We are not aware of other metagenome-analysis software that performs operon prediction or transport-reaction prediction. A number of other software tools Prakash and Taylor (2012); Huson et al. (2016) perform metabolic reaction and pathway prediction, often based on KEGG Kanehisa et al. (2021). The metabolic reconstruction approaches of KEGG and PTools differ in the following respects. They use different reference databases of pathways and reactions: as of August 2021, KEGG contained 400 metabolic pathway modules versus 2,969 metabolic pathways in the MetaCyc DB; KEGG contained 11,603 reactions versus 17,412 in MetaCyc. Thus, MetaCyc has far wider coverage of metabolism (7.4 times as many pathways, 1.5 times as many reactions). MetaCyc pathways were derived from and cite 69,000 literature citations and 9,739 textbook-equivalent pages of mini-reviews that explain the role of each pathway; KEGG contains very few citations or mini-reviews. The KEGG algorithm for reactome and pathway prediction has never been published to our knowledge, therefore its processing steps are unknown, whereas the PTools pathway prediction algorithm has been published Karp et al. (2011). KEGG does not produce organism-specific metabolic network diagrams, but it does have a series of global overview maps that span all KEGG pathways, thereby showing many pathways that are not present in a particular organism.

MAPLE Takami et al. (2016) also uses KEGG for metagenome pathway analysis. Its pathway prediction method is based on the “module completion ratio,” that is, assessing the evidence for pathway presence based solely on the fraction of reactions within a pathway that have an enzyme present. This simple method causes many false-positive predictions—particularly for larger pathway DBs such as MetaCyc—which is why we developed a more elaborate prediction method that considers factors such as pathway taxonomic range and key reactions Karp et al. (2011).

### 2.2 Searching Across an Organism Community

A suite of search tools enables scientists to perform basic searches across a set of microbiome-derived PGDBs, such as to determine which organisms in the community contain a given gene, protein, metabolite, or pathway. Such searches enable a researcher to quickly determine the functional roles played by different organisms in the community. In addition, more advanced searches are supported to find the organisms in the community containing genes, proteins, metabolites, or pathways matching specified conditions.

These searches are available in both the web and desktop modes of PTools, with somewhat different user interfaces available in the two modes. In web mode, the multi-organism search tools are present under the Tools > Search menu. For example, the Search Pathways command enables multi-organism pathway searches, the Search Genes, Proteins, or RNAs command enables multi-organism searches against genes and gene products, and the Search Compounds command enables multi-organism metabolite searches. By default these tools perform single-organism searches; to enable multi-organism searches, click the box next to “Search across Multiple Organisms/Databases.”

**FIGURE 1:**
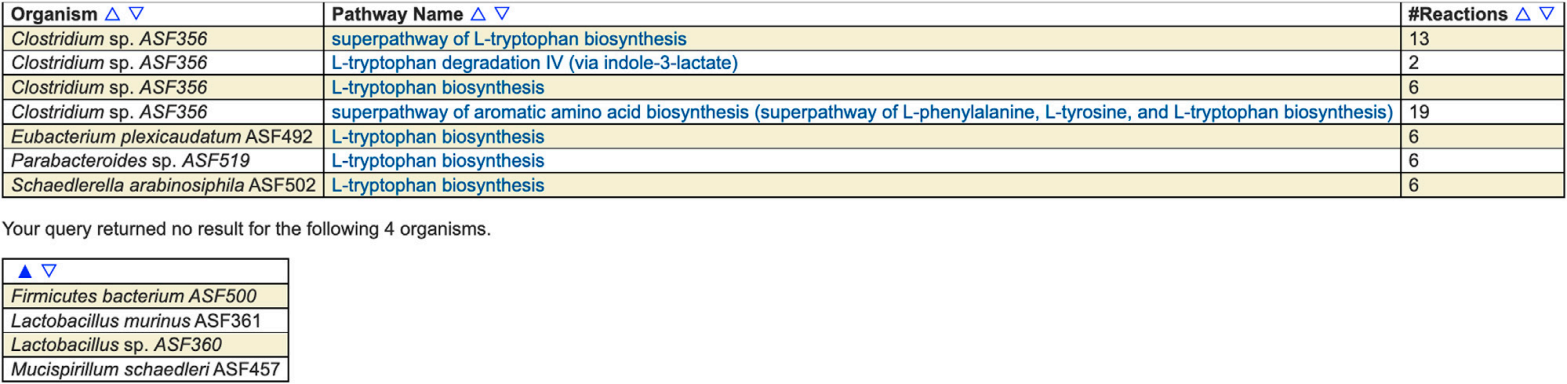
Results of searching across the ASF for pathways whose name contains “tryptophan.” Four of the organisms contain such a pathway and four do not. The pathways include biosynthesis and degradation, as well as super-pathways and base pathways.

For example, Figure 1 shows the result of searching across the ASF PGDBs on BioCyc.org for all pathways whose name contains “tryptophan.” Pathway searches can also search by ontology (such as for all detoxification pathways in the organism), pathway length, substrate(s) contained within the pathways, evidence code, and publication.

Gene/protein searches can search by sequence length, molecular weight, genome map position, pI, evidence code, cellular location, Gene Ontology (GO) term, publication, and by protein features.

Metabolite searches can search by ontology, monoisotopic mass, molecular weight, chemical formula, SMILES Anderson et al. (1988) substructure, and InChI Stein et al. (2003).

We are not aware of other tools that provide these types of multi-MAG search capabilities.

### 2.3 Comparative Analysis Operations on a Microbiome

**FIGURE 2:**
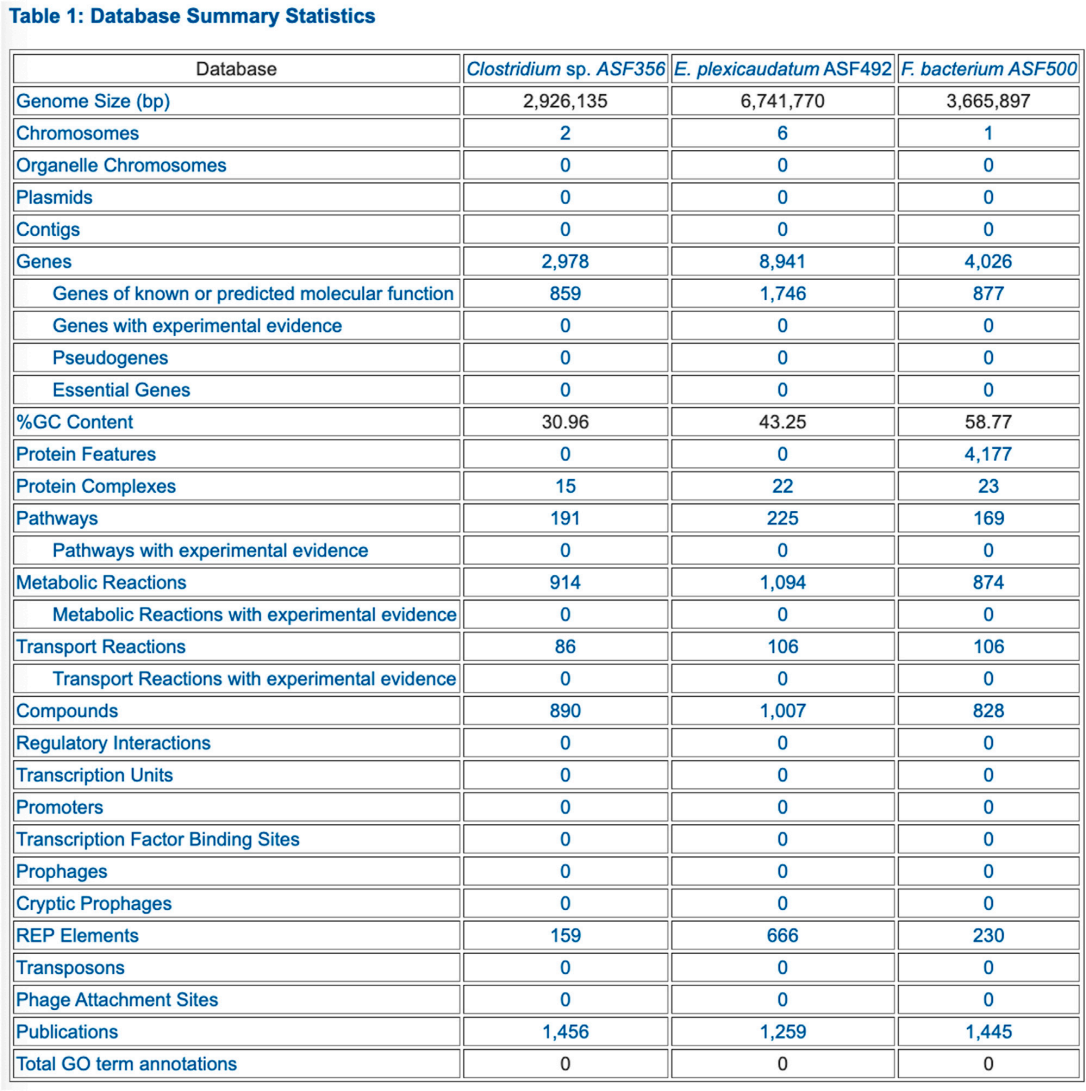
PTools generated table that summarizes database contents for three selected ASF organisms.

**FIGURE 3:**
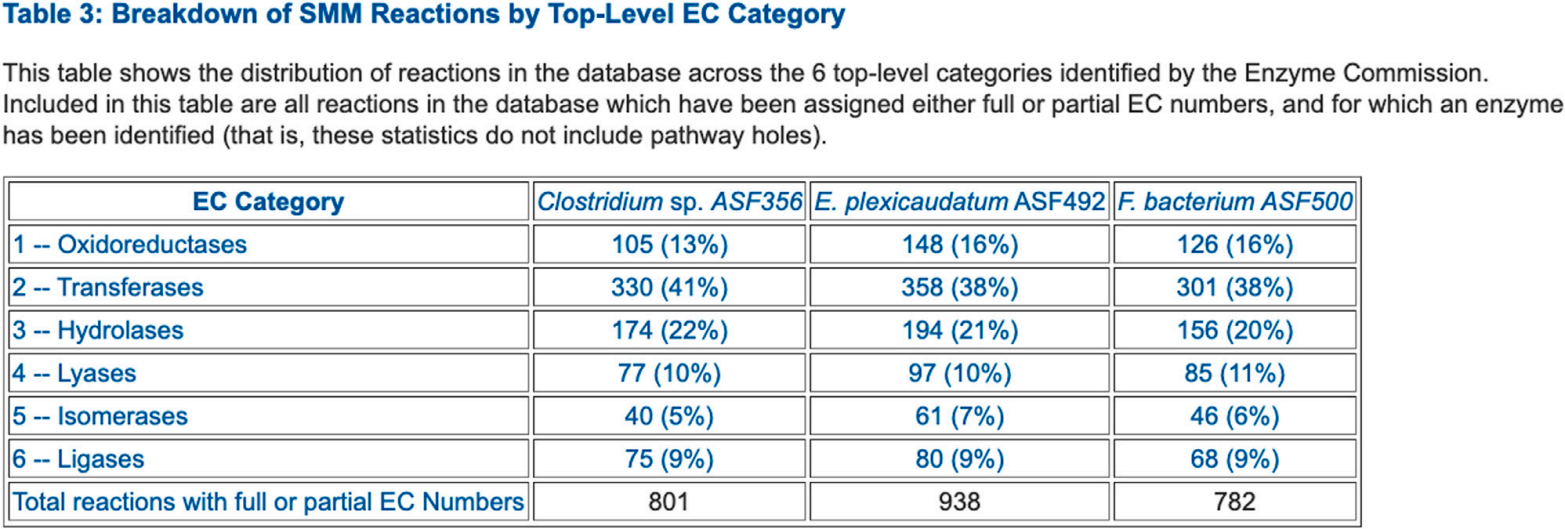
Table that summarizes the number of enzymes in each Enzyme Commission top-level category for selected ASF organisms.

**FIGURE 4:**
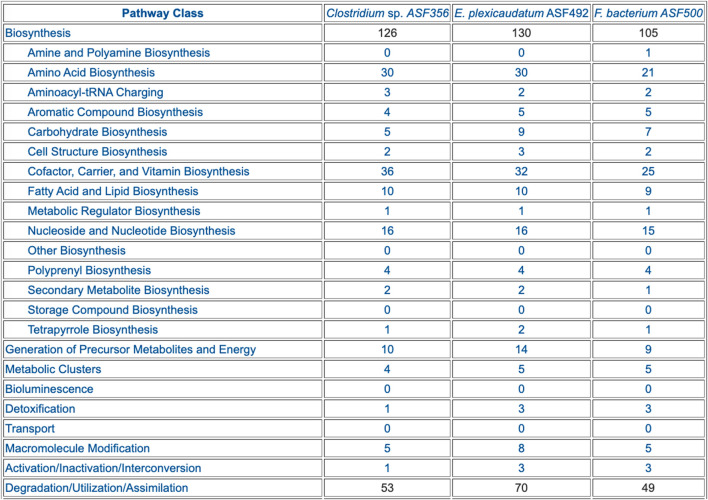
Table summarizing the pathway composition of selected ASF organisms, organized by the MetaCyc pathway ontology. The table is truncated for space considerations.

**FIGURE 5:**
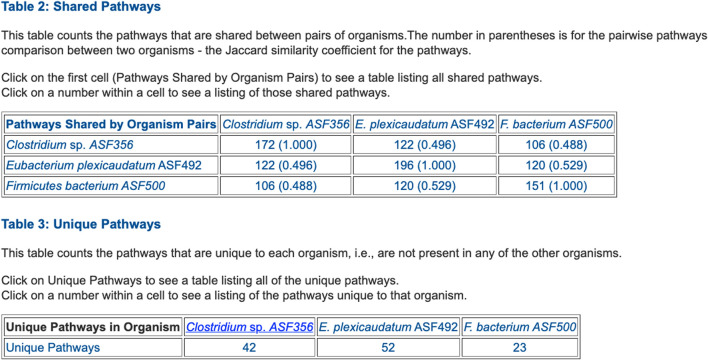
Table summarizing the number of metabolic pathways shared between pairs of selected ASF organisms, and the number of pathways unique to each of the three organisms.

**FIGURE 6:**
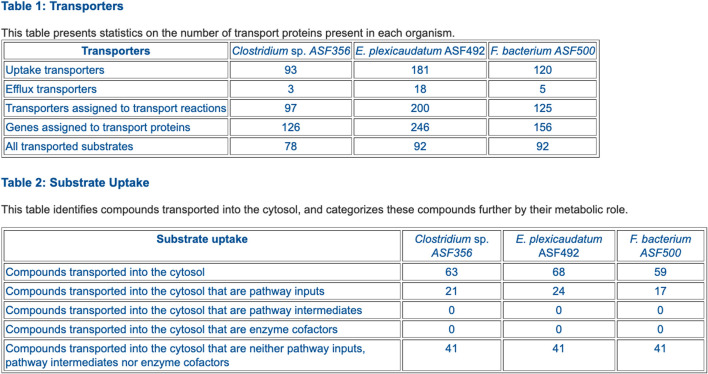
Table comparing the transporter complements of selected ASF organisms.

PTools provides an extensive set of comparative operations that can be run across a set of PGDBs for a microbial community. Each comparative operation generates a series of pre-defined tables. The comparative operations are available at BioCyc.org under Tools > Comparative Analysis. The comparison tables (some of which are appropriate for genomes, but not for MAGs) span these aspects of the selected PGDBs (table numbers refer to tables within the web pages):

• Organism comparison
  • Table 1: Database Summary Statistics (example in Figure 2)
  • Table 2: Phenotype Metadata
  • Table 3: Collection Metadata
  • Table 4: Annotation Metadata
• Reaction comparison
  • Table 1: Breakdown of Reactions by Type
  • Table 2: Reactions of Small Molecule Metabolism (SMM)
  • Table 3: Breakdown of SMM Reactions by Top-Level EC Category (example in Figure 3)
  • Table 4: Distribution of Isozymes across SMM Reactions
  • Table 5: Shared Reactions
  • Table 6: Unique Reactions
• Pathway comparison
  • Table 1: Breakdown of Pathways by Pathway Class (example in Figure 4)
  • Table 2: Shared Pathways (example in Figure 5)
  • Table 3: Unique Pathways (example in Figure 5)
  • Table 4: Pathway Holes
• Metabolite comparison
  • Table 1: All Compounds
  • Table 2: Shared Compounds
  • Table 3: Unique Compounds
  • Table 4: Statistics on the Frequency with which Different Compounds Appear in Different Metabolic Roles
• Gene/protein comparison
  • Table 1: Selected Gene/Protein Statistics
  • Table 2: Gene Annotation
  • Table 3: Frequency Distribution of Heteromultimers by Number of Unique Gene Products
  • Table 4: Enzymes
  • Table 5: Multifunctional Enzymes
  • Table 6: Gene Ontology
• Transporter comparison
  • Table 1: Transporters (example in Figure 6)
  • Table 2: Substrate Uptake (example in Figure 6)
  • Table 3: Substrate Efflux
  • Table 4: Multiple Transporters and Substrates
  • Table 5: Transcription
• Transcription unit and regulation comparison
  • Table 1: Number of Genes per Transcription Unit
  • Table 2: Number of Operons per Pathway
  • Table 3: Regulation

**FIGURE 7:**
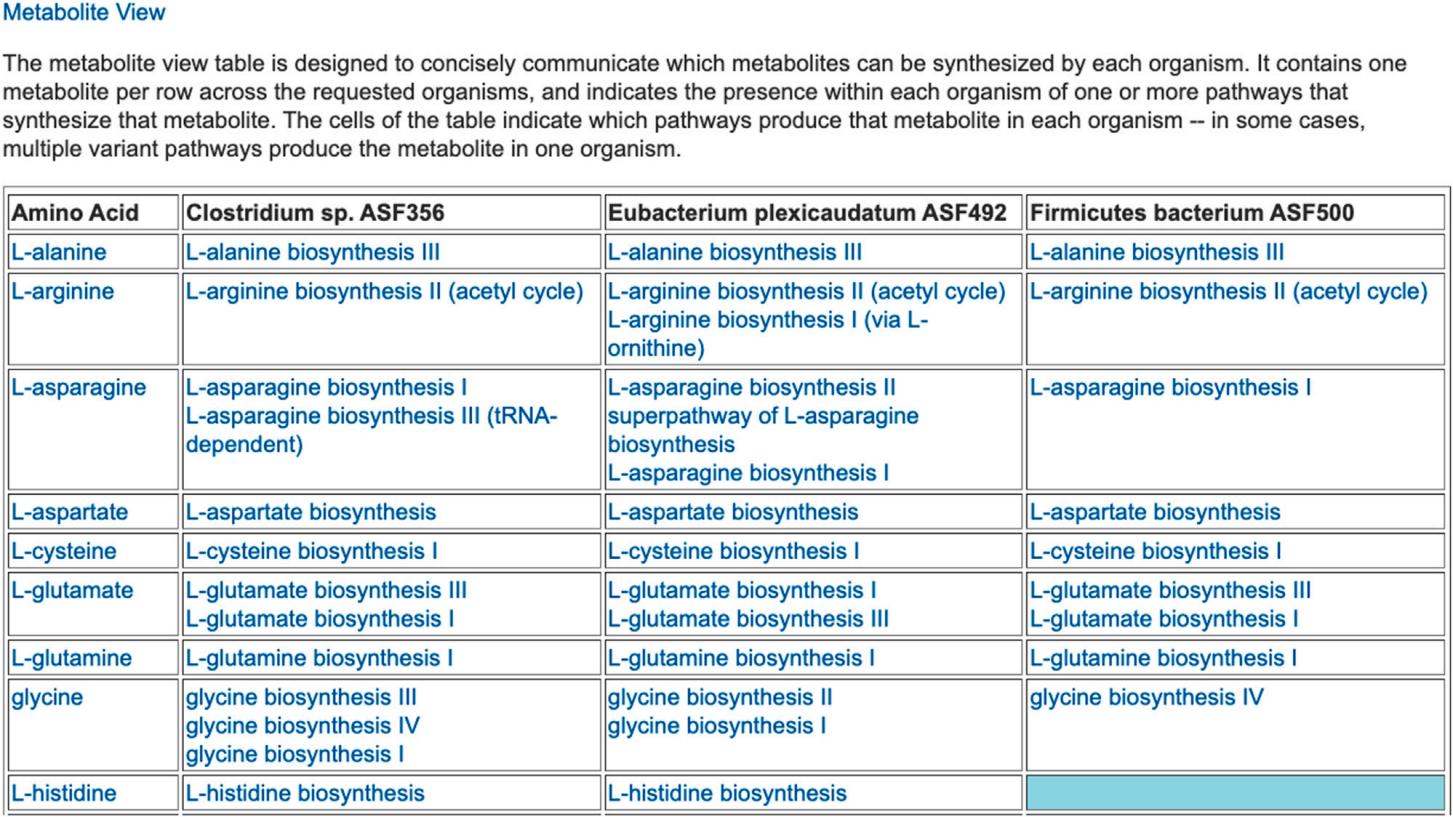
For each of three selected ASF organisms, this figure lists the biosynthetic pathways it contains for each amino acid. Multiple variants of amino-acid biosynthetic pathways are often known, as designated with roman numerals. The blue cell indicates that Firmicutes bacterium ASF500 does not contain a pathway for biosynthesis of L-histidine. The table is truncated for space considerations.

The preceding tables are computationally generated such that clicking hyperlinks within the tables will produce a new table with an expanded level of information. For example, clicking on the row name “Amino Acid Biosynthesis” in Figure 4 will generate the table shown in Figure 7, which shows the biosynthetic pathways present in each organism for each amino acid.

A number of other tools (e.g., MEGAN) present summaries of pathway abundances across different metagenome samples. In contrast, PTools reports differences in pathway compositions of different MAGs; we are not aware of other tools that perform such comparisons.

### 2.4 Analysis of Meta-Transcriptomics and Meta-Metabolomics Data

In a PGDB for a single organism, the PTools-generated cellular overview diagram provides a visual summary of all the metabolic and transport capabilities of the organism. A rectangular outer border represents the cell membrane. For Gram-negative bacteria, this consists of a double membrane with an intervening periplasmic space. Transporters and other membrane proteins are drawn on the appropriate membrane. Within the interior, representing the cytosol, metabolic pathways are shown to the left, and a grid containing all reactions not assigned to any pathway appears to the right. Within the pathway section, pathways are organized according to the MetaCyc pathway ontology, with biosynthetic pathways to the left, energy metabolism pathways in the middle, and catabolic pathways to the right. These sections are further subdivided into functionally-based blocks. For example, within the biosynthetic section are separate blocks for Carbohydrate Biosynthesis and Secondary Metabolite Biosynthesis. Pathways generally flow downwards, and connections between pathways are mostly omitted. As the user zooms in on the diagram, more detail is shown. At the highest level of detail, pathway, metabolite, enzyme and gene names all become visible. Users can overlay omics data for an organism onto the cellular overview diagram to visualize experimental results in a metabolic context Paley et al. (2021).

For a community of organisms, the user can create a community overview diagram (within the desktop version of PTools only) that condenses and combines the overview diagrams from multiple organisms into a grid, forming a single large diagram. While initially shown at a low level of detail, users can interrogate the diagram via mouse-overs, zoom in to show more detail, or apply a range of highlight operations. Meta-transcriptomics or meta-metabolomics data can then be mapped onto this community overview diagram to visualize how experimental conditions affect the metabolism of the entire community. Omics data are supplied as a set of tab-delimited files, one per organism in the community, each with the first column containing gene or metabolite identifiers, and a single numeric data column (any additional columns in the file will be ignored), which can contain either absolute data (e.g., counts, intensities, concentrations) or relative data (e.g., ratios or log ratios of two experimental conditions or experiment *vs*. control).

**FIGURE 8:**
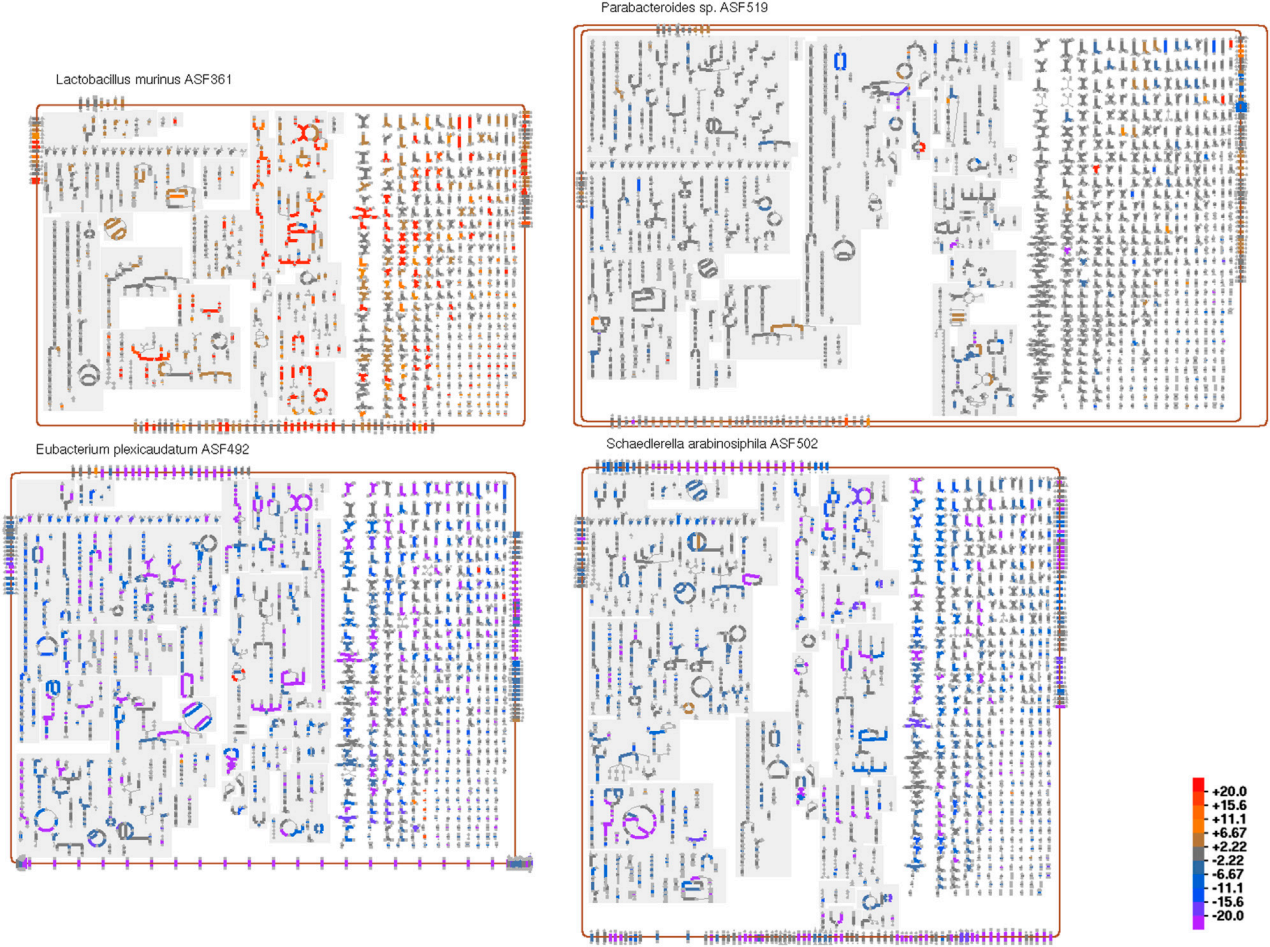
A community overview diagram for four of the bacterial species that make up the Altered Schaedler Flora model gut microbiome, overlaid with data from an example transcriptomics dataset. Reactions colored orange or red indicate genes with increased expression levels, whereas reactions colored blue or purple indicate genes with reduced expression levels.

Figure 8 shows a community overview diagram consisting of four organisms from the ASF microbial community overlaid with an example transcriptomics dataset. To identify the metabolic pathways that showed differential activity in response to altered gut environmental conditions, we conducted global transcriptome analysis (RNA-seq) of the ASF community recovered directly from wild type mice (129Sv6 background) along with IL-10^−/−^ knockout mice on the same genetic background. IL-10 is a well-characterized immunomodulatory cytokine and IL-10^−/−^ knockout conventional (i.e., complex microbiota) mice are known to exhibit an altered microbiota composition Overstreet et al. (2021). Figure 8 shows the functional changes in the microbiome as the ASF responded to the altered immune status of the host as determined by identifying differentially expressed genes associated with specific metabolic pathways. The transcriptome dataset used for this analysis was generated by DeSeq2 Love et al. (2014).

In addition to visually drawing attention to particular metabolic reactions and pathways that undergo significant change, organism-wide effects also become apparent. For example, in this dataset we immediately notice that the metabolism of one organism, *Ligilactobacillus murinus* (i.e., *Lactobacillus murinus*) is generally increased (red/orange reactions), with the increases concentrated in certain pathway classes; the metabolism of two other organisms, *Eubacterium plexicaudatum* and *Schaedlerella arabinosiphila*, generally decreases (blue/purple). Mousing over any reaction will show a tooltip that includes the omics data values for all genes associated with that reaction. The user can zoom in on the diagram for a more detailed view of regions of interest.

We are not aware of other tools that can display metabolic network diagrams from multiple organisms simultaneously and paint these diagrams with meta-omics data.

### 2.5 Community Metabolic Route Search

Single-organism metabolic route search enables the discovery of the most optimal series of reactions (called routes), that will transform a starting compound into a goal compound, within the organism’s reaction network. Optimal means that the reaction series has the lowest cost. The cost of a route is computed by a weighted combination of atom conservation, route length in terms of sequential reactions, and other parameters. To compute the number of conserved atoms, our RouteSearch algorithm Latendresse et al. (2014) uses pre-computed atom mappings Latendresse et al. (2012) of reactions that are available in MetaCyc. An atom mapping of a reaction gives a one-to-one correspondence of each non-hydrogen atom, from reactants to products. The more atoms are conserved, the more efficient the transformation from start to goal becomes, thus resulting in a lower cost.

The Multi-Organism RouteSearch (MORS) algorithm Krummenacker et al. (2019) is a recent extension of single-organism RouteSearch that enables route discovery across arbitrary sets of organisms, simultaneously searching across the union of reactions in their PGDBs. MORS enables dissecting the metabolic contributions originating from specific organisms, within the overall transformation performed by the microbial community. A typical use case is searching HumanCyc as well as the organisms in a microbiome body site, such as the gastrointestinal-tract, to investigate how a combination of organisms might synthesize a compound that is toxic to the host organism. To perform MORS searches at BioCyc.org, invoke Tools > Metabolism > Metabolic Route Search, and check the box next to “Routes across Multiple Organisms.”

The MORS algorithm requires an additional input beyond the inputs to RouteSearch, namely the set of PGDBs to be searched. The reaction network searched by MORS will be the union of all reactions from that organism set. Additionally, the user may alter a new MORS parameter, the cost for “organism switching.” A switch occurs when the two organism sets of two consecutive reactions in a route have no overlap. In other words, if the first reaction is known to occur in one set of organisms and the second reaction is occurring in a different organism set, but there is no organism that contains both reactions simultaneously, then the route must switch organisms by transferring the compound connecting both reactions from one organism to another (by unspecified transport mechanisms). Each discovered route is displayed horizontally across the web page, with the start compound on the left and the goal compound on the right. An organism switch is depicted in a route by a red vertical line. A SmartTable of the route can be generated, which lists the organism sets that provide the enzymes that catalyze each reaction along the route.

**FIGURE 9:**
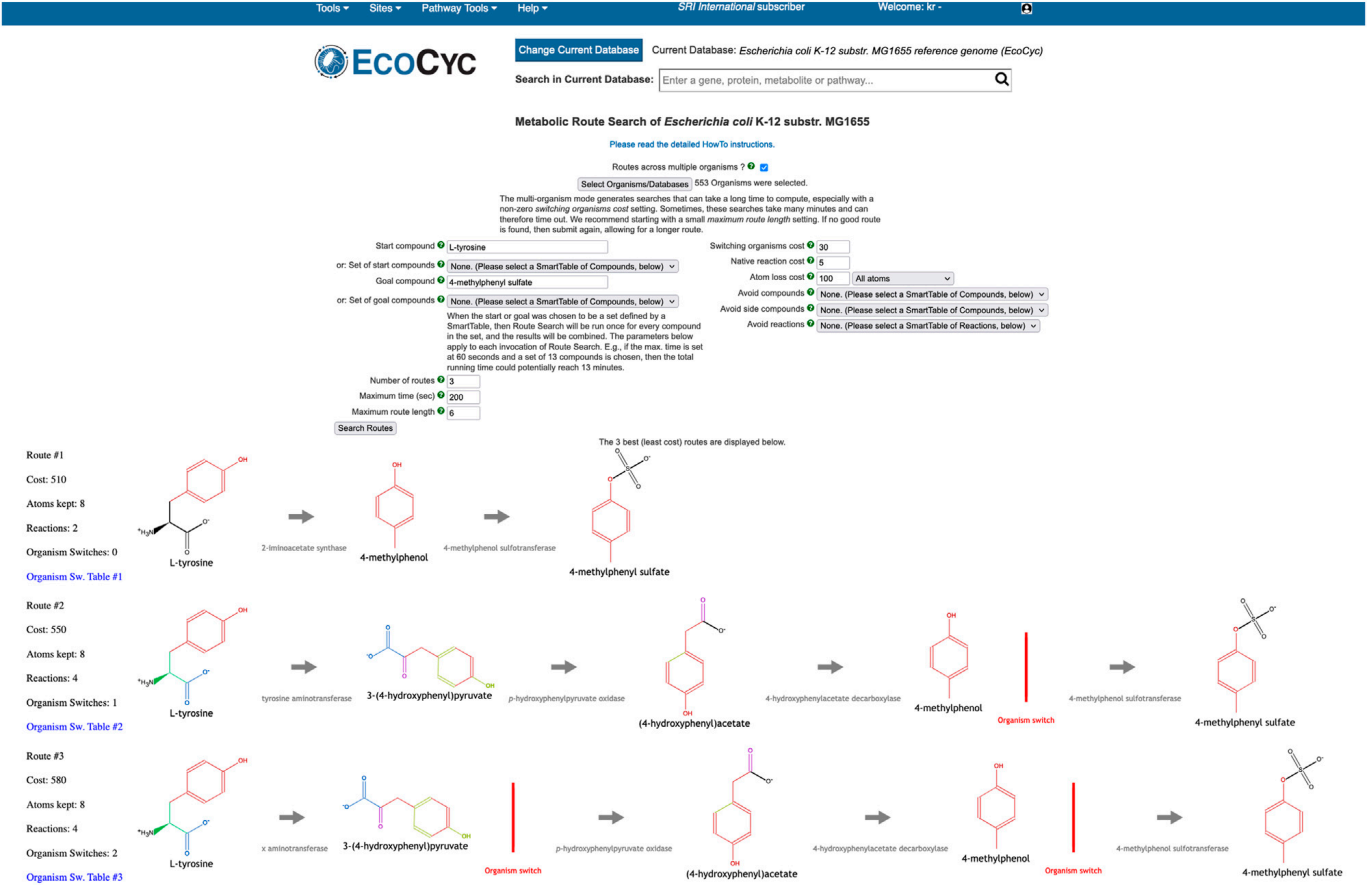
MORS computed routes from L-tyrosine to 4-methylphenyl sulfate.

As an example, let us use BioCyc.org to examine how dietary L-tyrosine is transformed into toxic 4-methylphenyl sulfate, which is a protein fermentation product that has been modified in the liver and is implicated in kidney disease. As it is known that this toxin originates from L-tyrosine Selmer and Andrei (2001), the MORS start compound was set to L-tyrosine and the goal compound to 4-methylphenyl sulfate. We selected all organisms in the human microbiome body site called “gastrointestinal-tract” plus *Homo sapiens*. The total count of organisms was 553. The resulting top three routes are shown in Figure 9. All routes retain eight atoms. The first route consists of two reactions, and the other two routes consist of four reactions. The first route does not need an organism switch, because one microbe was found that can perform both reactions of this route. In the other two routes, the last reaction after the organism switch is found only in *Homo sapiens*. However, the reaction immediately before the switch occurs in 26 organisms in both routes. The third route found a different choice for the first reaction, which incurs the cost of an additional organism switch.

We are not aware of other tools that can perform multi-organism metabolic route search.

## Data Availability

Publicly available datasets were analyzed in this study. This data can be found here: biocyc.org.
